# What Determines the Acceptance and Use of eHealth by Older Adults in Poland?

**DOI:** 10.3390/ijerph192315643

**Published:** 2022-11-24

**Authors:** Justyna Rój

**Affiliations:** Department of Operational Research and Mathematical Economics, The Poznań University of Economics and Business, Al. Niepodległości 10, 61-875 Poznań, Poland; justyna.roj@ue.poznan.pl

**Keywords:** eHealth, older adults, nested regression analysis, UTAUT model

## Abstract

An aging population is considered a major challenge for governments and healthcare planners. eHealth is perceived as a tool with the potential to ensure efficient healthcare. Moreover, eHealth services may help older adults to maintain longer life in good health. However, there are still several challenges to the large-scale implementation of these solutions among older adults. Therefore, the aim of this study was to explore determinants of the acceptance and use of eHealth by older adults in Poland. Data was collected by the questionnaire, and the UTAUT model was employed. This research covered older adults aged 60 to 69. The analysis of the results using nested regression analysis showed that performance expectancy has a strong significance on the older adults’ acceptance and use of eHealth, followed by effort expectancy and social influence. In contrast, facilitating conditions do not have a significant influence on the acceptance and use of eHealth. These findings may also be beneficial for the government to provide relative policies to support the development and usage of eHealth services as well as for the healthcare devices industry to design more older adult-oriented products.

## 1. Introduction

Everywhere in the world, people are living longer. In the European Union, life expectancy at birth for females is expected to increase by 6.1 years from 84.2 in 2019 to 90.3 in 2070, and it is projected to increase for males by 7.4 years over the projection period from 78.7 in 2019 to 86.1 in 2070 [1]. Moreover, over the next three decades, the global number of older persons is going to double [2]. Such increase in life expectancy is due to advances in healthcare as well as in economic and social development, which contribute to the control of disease, prevention of injury, and reduction in the risk of premature death, and accounts for improvements in survival at older ages and thus the overall improvement in longevity. It reflects positive human development, but it also creates both economic and societal challenges [3]. As more people are living longer, they would like to stay healthy and active to fully participate in life [4]. However, when these additional years of living are dominated by biological and cognitive degeneration inherent to aging, then it may limit full social, cultural, and intellectual engagement and would have negative implications for the value of their life as well as for the whole society [5]. So, the increasing demand for primary health care and for long-term care is a real challenge for healthcare systems. Therefore, the United Nations General Assembly declared 2021–2030 the Decade of Healthy Aging, which means “the process of developing and maintaining the functional ability that enables wellbeing in older age” with the World Health Organization (WHO) as the leader of this plan [6].

It was noticed that information and communication technologies (ICTs) could play a significant role in facilitating active and healthy aging [7]. Such secure and cost-effective use of ICT in support of health and health-related fields, including health-care services, health literature, and surveillance, as well as health education, knowledge, and research, is referred to as e-Health [8]. In other words, eHealth is “the electronic exchange of health-related data collected or analyzed through electronic connectivity to improve the efficiency and effectiveness of healthcare delivery” [9]. There are also both theoretical and empirical studies that provide evidence that eHealth has the potential to improve health outcomes by enhancing diagnostic procedures, data-based treatment decisions, clinical trials, digital therapeutics, self-management of care and person-centered care, creation of competence and skills for professionals to support health care [10,11]. Therefore, many governments and healthcare organizations started to perceive eHealth as one of the important elements in solving the problem of the increased demand for healthcare by the aging population [12] and a key factor in improving their wellbeing [13] at least by allowing older adults to increase their control over their disease(s) and their own condition [14]. Indeed, the majority of empirical studies on eHealth and healthy aging showed positive effects of these interventions for physical dimensions [15,16,17], social dimensions [18,19], and cognitive dimensions of older adults’ health [20]. 

On the other hand, it was also recognized that too great a reliance on eHealth has the potential to increase disparities between those who have skills and access to digital tools and those who do not [21]. Finally, eHealth could increase health disparities, whereas equity is one of the important values of healthcare systems as it applies to fair opportunity for everyone to achieve their full health potential regardless of demographic, social, economic, or geographic status [22]. Thus, it implies the minimization of differences in access, coverage, quality, use, and utility of healthcare services between groups of the population classified by the above characteristics [23]. 

Such concerns about a potential increase in disparities exist in particular in the case of older adults as they appeared to be slower to adopt new technologies compared to younger adults [24]. They are still not familiar with ICT solutions and do not use them to the same extent as other generations do, as they face more difficulties with learning new software [25,26,27,28,29]. It is often attributed to age-related cognitive decline [30,31]. ICT learning relies on skills that are associated with fluid intelligence and may therefore differ between younger and older people [32]. In addition, some empirical studies showed that the low level of IC technology usage by older adults is related to extreme and irrational fear and anxiety about managing digital tools. Technophobia is recognized as a possible new risk factor for older adults because it can affect their life through low conformity to digital living [33,34].

However, one of the most important factors for the success of health information technology implementation is users’ acceptance of that technology [35]. Technology’s features play a vital role in determining whether individuals involved in an activity will use it or not. Therefore, understanding the users’ perception toward the adoption of new technology could help facilitate further growth of the implementation of that particular technology [36]. Such understanding is vital to enable the interested parties, such as academics and practitioners (among others: researchers, government, organizations, and businesses), to relate with both the theory and practical aspects of the technology acceptance models and theories [37]. Academicians are interested in realizing the factors that drive users’ acceptance or rejection of technologies while recognizing the needs and acceptance of individuals is the beginning stage of any business, and this understanding would be helpful in finding the way to future development [38].

Therefore, understanding the factors affecting the acceptance and use of healthcare technologies is a crucial topic that has been extensively studied, specifically during the last decade [39]. The literature review showed that there are many available technology acceptance theories (models) that focus on the technology factors, which emphasize the attitude, perception, and interaction between humans and technology [40]. Such models also allow us to identify the barriers related to ICT adoption by older adults as well as elements supporting their attitudes and perceptions toward ICT use [41,42,43].

The analysis of systematic review articles published on technology acceptance in healthcare also showed that the Technology Acceptance Model (TAM) and Unified Theory of Acceptance and Use of Technology (UTAUT) models are the most prevalent models in explaining what affects the acceptance of various healthcare technologies through different user groups—health-care professionals and patients (also older adults), settings, and countries [39,44,45,46,47]. Moreover, nowadays, the UTAUT model is still one of the commonly-used theories for technology adoption studies in various other areas as well as in the medical sector [39,47]. In fact, internationally, this methodology has been applied many times in surveys conducted in the healthcare sector, confirming the acceptability of the UTAUT model in different applications of eHealth by also taking different perspectives, either health centers [35] or healthcare professionals [48,49,50,51,52,53] or both patients’ and health professionals’ acceptance [54] or general users of an eHealth [55], including older adults [56,57,58,59]. However, based on the systematic review articles on older adults and eHealth [60,61,62,63] and the abovementioned quantitative studies, it was found that these studies differ in the range of users, their scope, sample size, location, and the type of health ICT being verified (telecare, telemedicine, telemonitoring, health monitoring devices). 

So, in Poland, the use of ICT has expanded rapidly in the last decade, but knowledge about the acceptance and use of eHealth within the population of older adults is still scarce. However, all over the world and in Poland as well, we can observe the fast development and use of ICT in many different areas of life. Poland is one of the European Union countries and is located in Central Europe, with a population of approximately 38.2 million as of 2021, which makes it the ninth-most populous country in Europe and the fifth-most populous member state of the European Union [64]. Poland also faces contemporary healthcare challenges, including aging of the society and medical staff shortage [65] which are likely increasing demand for eHealth solutions. The study of [66] confirmed the word tendency in Poland that the number of illnesses increases with age. Moreover, [67] showed that the demand for medical procedures and hospitalizations among senior citizens is the highest compared to other age groups. 

However, [68] found that the older adult patients surveyed in Poland are not overly enthusiastic about using information and communications technology tools in their healthcare. Ref. [69] also showed that even though eHealth is a fast-growing area of healthcare in Poland as its development is strongly supported by the European Commission (EC), it is not effectively supported by the central government and the National Health Fund (the public purchaser of health services in Poland). Another study of [70] showed that doctors were quite pessimistic when they were asked about the possible use of telemedicine among senior citizens in Poland. However, [66] provided evidence that about 40% of the Polish seniors surveyed (312 persons) aged 60–79 declared a willingness to use definite telemedical services. Moreover, the study showed that seniors with constant access to mobile devices (the Internet and mobile phones) are more inclined to regard telemedicine as necessary. Later, [71] found that older people (a group of 363 respondents aged 60+) in Poland are more and more efficient when using e-health services due to their increasing ICT competencies. However, [72] recognized the existence of many barriers to telemedicine’s development in Poland, such as legislation and an underdeveloped system of healthcare finance, but especially a low level of awareness of the system’s participants in all age groups. Moreover, [73] found that awareness is the main barrier to the implementation of telemedicine services for older adults. Then, empirical analysis of [74] highlighted that the older adults are often resistant to use telemedicine. The UTAUT model was applied only by [53] to identify those aspects of the e-prescription system in Poland that best-facilitated doctors’ willingness to use it. Moreover, [75] also applied the UTAUT model in their research on the intention of generation Z to use online health information. However, no study on the acceptance and use of eHealth, particularly concerning older adults, has been conducted in Poland.

Therefore, to fill in this gap and being inspired by the research of [76], this survey applied the original Unified Theory of Acceptance and Use of Technology (UTAUT) model to investigate factors that influence the acceptance and use of eHealth by older adults, given the complex environmental, cultural, and social paradigm in Poland. To the authors’ best knowledge, no prior review has been specifically examined. Thus, it raised the question: what determines the acceptance and use of eHealth by older adults in Poland? 

The aim of this study was to explore determinants of acceptance and use of eHealth by older adults in Poland. This research covers one sub-group of older adults, i.e., aged 60 to 69, and is based on the constructs of the UTAUT paradigm. 

Thus, this survey study creates a framework and explores determinants of the acceptance and use of eHealth by older adults. As there is no similar research in the context of Poland, this study believes that the application of the UTAUT model makes a significant contribution to the literature and healthcare policy in that it is the first one to investigate the factors influencing the acceptance and use of eHealth by older adults in Poland. Therefore these findings may also be beneficial for the government to provide relative policies to support the development and usage of eHealth services as well as for the healthcare devices industry to design more older adult-oriented products. Consideration of the specific barriers and facilitators that influence the acceptance and use of eHealth by older adults is critical to improving their use of eHealth programs.

This study is structured as follows: introduction contains the theoretical background and the aim of this study, and they are provided in Section 1; Section 2 introduces the data and methods used in this article; the results are shown in Section 3, and the theoretical and management implications are shown in Section 4; in Section 5, the conclusions of this article were drawn.

## 2. Materials and Methods

### 2.1. Participants

The study involved the population of older adults aged between 60 and 69 and consisted of a sample of 400 older adults (in the mentioned range of age) who were participants of the research panel “Badanie Opinii” run by Biostat (note: Biostat—the research and development center, which has been supporting the medical environment and pharmaceutical industry for 14 years by providing comprehensive solutions in the field of statistics, scientific research, and medical applications as well as a high-quality data (http://www.biostat.com.pl, accessed on 10 October 2022). “Badanie Opinii”—research panel provides the opportunity to regularly examine preferences, opinions, etc., and it covers wide range of panelists (consumers)). Data was collected in January 2022. At that moment, there was total of 5,185,843 older adults aged between 60 and 69 in Poland. Following the United Nations’ definition of older adults and WHO’s approach in the context of healthy aging, the age of 60 was chosen as a starting point. Then, the age of 69 was selected as the cut-off point due to the cost and technical constraints of this research and to ensure that the sample is representative. 

It was possible to draw the sample of older adults between the age of 60–69 in such a way that they are representative in terms of gender and then voivodeship (geographical location). The voivodeship (province) is the highest-level administrative division of Poland, corresponding to a province in many other countries. The territory of Poland is administratively divided into three levels of subdivision. First, Poland is divided into provinces (voivodeships), which are further divided into powiats (counties or districts), and these, in turn, are divided into gminas (communes or municipalities). At the present moment, Poland has 16 provinces.

Thus, the sample included a representative distribution by gender and provinces (voivodeship) in relation to the population of Poles at the analyzed range of age. In case of age—the total population of older adults aged between 60 and 69 in Poland—this means 5,185,843 people—consists of 54% females and 46% males. Therefore, the analyzed sample also includes 54% females and 46% males. Moreover, an analogous approach was applied in the context of provinces.

### 2.2. Measures and Procedure

This research employed the UTAUT model, which was formulated by Venkatesh et al. (2003) [77]. It aimed to combine usage models by analyzing, reviewing, and integrating constructs from eight competing models. These models are: theory of reasoned action (TRA) [78], motivational model (MM) [79], theory of planned behavior (TPB) [80], model of PC utilization (MPCU) [81], social cognitive theory (SCT) [82], innovation diffusion theory (IDT) [83], a combined theory of planned behavior/technology acceptance model (C-TPB-TAM) [84], and also technology acceptance model (TAM) [85].

In this way, repetitions and redundancy were eliminated as many constructs in these theories were held in common [86]. Thus, UTAUT outperformed all eight models using the same data offering stronger predictive power compared to the rest of these models that examine technology acceptance [87,88,89,90]. Therefore, given the complex environmental, cultural, and social paradigm in developing countries, UTAUT is perceived in the technology acceptance literature as a significant step forward [91].

UTAUT postulates four core predictors of the acceptance and use of technology and which are treated as independent variables within the model:

(1) performance expectancy (PE), which is defined as the degree to which an individual believes that using the system will help him/her to attain gains or receive benefits in health status;
(2) effort expectancy (EE) means the degree of ease associated with the use of the system”, as users tend to consider the effort required before using the information system.“
(3) social influence (SI) is the degree to which an individual perceives that important others (family members, friends, or some other people who are an authority for us like a peer group) believe he or she should use the new system; as the preferences and values of society tend to change the viewpoints of users profoundly;
(4) facilitating conditions (FC) is defined as the degree to which an individual believes that an organizational and technical infrastructure exists to support the use of the system [77].

Moreover, four possible moderating variables are proposed, such as: gender, age, experience, and voluntariness of use which are treated as the independent variables. The first models and theories connected with technology acceptance did not take into account the above moderators [87,88]. It is the advantage of UTAUT model as other studies prove that models that include moderators often provide a significantly better explanation of technology acceptance or avoidance but also for this reason UTAUT model has often been criticized as too complicated for research. UTAUT model also faced some criticism with regards to its inability to explain acceptance and use of technology in different settings [91]—thus focusing on a single subject—in terms of a community, culture, country, organization, agency, department, etc., and it was the most widely reported constraint [39,55]. In [92], the UTAUT model was criticized as being less parsimonious than the previous TAM and TAM2 because its high (R^2^) is only achieved when moderating key relationships with up to four variables. They also called the grouping and labeling of items and constructed problematic because a variety of disparate items were combined to reflect a single psychometric construct. According to [93], the UTAUT might be a powerful model due to its parsimonious structure and higher explanatory power (R^2^). However, he criticized this model as it did not examine direct effects, which might reveal new relationships and important factors from the research which were left out by subsuming under the existing predictors only [94].

Data were collected by using the questionnaire. The questionnaire allowed the collection of the respondents’ demographic profiles like gender, education, and geographical location. This questionnaire surveyed the respondents’ attendance and use of eHealth and was constructed based on the element of the UTAUT model and especially based on [76], who has already successfully used it.

The questionnaire started with a description of the eHealth term. eHealth was described as the care obtained through the use of the Internet and regardless of the employed device, i.e., computer, tablet, and smartphone, such as: (1) arrangement of medical visit with a healthcare professional; (2) asking the healthcare professional a question via the internet; (3) e-consultation via the Internet; (4) obtaining medical care (treatment) or support via telecare from a doctor (5) measuring—for example—your blood pressure, blood sugar level at home and sending these results to a doctor as well as (6) face-to-face contact with a doctor via the Internet.

Then, the description of eHealth was followed by five general, pre-structured, multiple-choice questions which applied to the acceptance and use of eHealth (AU) and then four elements of the UTAUT model, which are expected to influence the AU: (1) performance expectancy (PE), (2) effort expectancy (EE), (3) social influence (SI), (4) facilitating conditions (FC). In the case of the facilitating conditions, the research focuses on Internet self-efficacy, which means the person’s belief that he or she is able to successfully use the Internet [76]. This Internet self-efficacy has been identified as a facilitating factor predicting the use of eHealth [76,95]. Thus, the respondents were asked to answer such specific questions and rate specific statements that correspond to the above elements of the UTAUT model. The first question is a measure of AU, and questions 2–5 are measures of PE, EE, SI, and FC, respectively.

(1) “Do you use or would you use one of the above- mentioned Internet applications (eHealth forms) in the future if you were offered the opportunity?”—respondents were supposed to choose one of the possible answers: (a) “yes, definitely”, (b) “yes, probably”, (c) “I don’t know”, (d) “no, probably not”, (e) “no, definitely not”. Then to each answer the appropriate score was assigned, starting with 5 for “a” and ending with 1 for “e”.
(2) Four statements (a–d) were used as the measures of performance expectancy: “Is your opinion contacting doctor via the Internet (a) makes it easier to contact a doctor when it is needed (b) it makes possibility for me to live longer (to facilitate disease prevention and regular health monitoring) (c) it works (functions) well, (d) it is a pleasant way to interact”. Respondents were asked to rate each statement, and the possible answers were provided as follows: “strongly agree” (score = 5), “agree” (score = 4), “I don’t know” (score = 3), “disagree” (score = 2), “strongly disagree” (score = 1). Then, the average of the scores on these four statements were taken as the score to express the performance expectancy.
(3) Three statements (a–c) were: “Is your opinion, contacting a doctor via the Internet (a) is easy to learn, (b) fits easily into my daily routine, (c) is easy to do.” The possible answers and ratings were used as in the case of statements in point 2. Moreover, the average of the scores on these three statements was taken as the score to express the effort expectancy.
(4) The following statement was asked to be rated by respondents: “Contacting a doctor via the Internet is something my family or friends do or would like to do”. The same categories of possible answers were given for statements in points 2 and 3.
(5) The question was: “How easy or difficult do you find it to use the Internet”. The possible answers were “very difficult” (score = 1), “difficult” (score = 2), “neutral” (score = 3), “easy” (score = 4), “very easy” (score = 5), “I don ‘t know; I don’t use the Internet” (score = 0).

### 2.3. Statistical Analysis

The measurement model was assessed by examining the internal reliability, convergent, and discriminant validity [96]. The internal reliability was evaluated considering Cronbach’s alpha and composite reliability, where the level of 0.70 is an indicator of acceptable internal consistency [97]. Convergent validity was tested by an average variance extracted (AVE) with at least 0.50 of AVE for construct validity [96]. The discriminant validity was assessed by the square root of the AVE and cross-loading matrix. The square root of the AVE of a construct should be greater than its correlation with other constructs for satisfactory discriminant validity [96].

In this research, both the descriptive statistics and Pearson’s r correlation coefficient were engaged. Moreover, a nested linear regression analysis was employed to identify and analyze the relationship between the acceptance and use of eHealth and the fourth element of the UTAUT model. Gender and education were used as background variables. Thus, the nested linear regression was conducted starting with the background variables such as gender and education (Block 1), and then the rest of the variables mean: performance expectancy (Block 2), effort expectancy (Block 3), social influence (Block 4), and self-efficacy (final model) block-wise were added to the model. All analyses were performed using Excel, STATA 13.3., and IBM SPSS Statistics 28.0.1.0 (142).

## 3. Results

The demographic characteristic of respondents showed that 216 females (54%) and 184 males (46%) participated in the study. Most of the participants (53.25%) had attained at least a medium education level, then 33.50% of them had high education while 11.25% were with vocational education, and 2% of them had a low (elementary) education level. All people are users of the Internet.

The analysis of correlations (Table 1) showed that there were strong relations between acceptance and use of eHealth and performance expectancy, and moderately strong with effort expectancy and social influence (all values of Pearson’s *r* were between 0.44–0.54 with *p* < 0.05), while weak with the facilitating conditions (0.25; *p* < 0.05). The relations between the following variables, such as: performance expectancy, effort expectancy, social influence, and facilitating conditions, were found to be strong and moderately strong, except for the relations between facilitating conditions with performance expectancy and social influence, which appeared to be weak. 

The assessment of measurement model validity confirmed its internal consistency, and conditions for the convergent and discriminant validity of data are satisfied in this study. The internal reliability was evaluated considering Cronbach’s alpha and composite reliability. The calculated Cronbach’s alpha values were 0.75 for PE and 0.81 for EE, and composite reliability ranged from 0.87 for PE to 0.84 for EE, which supports strong internal reliability. Then, in the case of PE and EE, construct loading ranged from 0.76 to 0.90, and the values of AVE were as follows: 0.631 for PE and 0.633 for EE, which means that they were greater than the recommended levels. Therefore, the conditions for convergent validity are satisfied in this study. 

The discriminant validity was assessed by the square root of the AVE and cross-loading matrix. The values of the square root of AVE were 0.794 for PE and 0.796 for EE and were greater than their correlation with other constructs for satisfactory discriminant validity. 

Table 2 shows the structure of respondents’ answers regarding the acceptance and use of eHealth. It was found that 72.50% of respondents declared the acceptance and use of eHealth. The gender distribution of responses is comparable.

Then, Table 3 presents the structure of the answers to the four questions of the questionnaire (2–5), which are related to the fourth element of the UTAUT model. More than half of respondents perceived the usefulness of eHealth, which facilitates contact with the doctor when it is needed and regular health monitoring with disease prevention. They also found it a nice way to contact the doctor. However, most of the respondents were not convinced about how well it works (57.75%). Generally, most of them had positive expectations and opportunities regarding the performance of using eHealth.

Regarding effort expectancy, most of the respondents found eHealth as easy to use and apply. Contacting a doctor via the internet was perceived by 83.50% of surveyed older adults as easy to learn and by 78.25% of them as easy to do, while for 65.25%, it also fitted easily into their daily routine.

Almost one-third of respondents did not know whether their important others (in this case: friends or family) would use eHealth or not. Moreover, one-fourth of respondents’ friends and family did not tend to use eHealth. Then, 82.75% of respondents found using the Internet easy or very easy. As internet self-efficacy has been used as a facilitating factor (facilitating condition), it means that most of them have the necessary support to use eHealth. 

Regression analysis (Table 4) showed that the background characteristics (gender and education level) only explained 2% of the variation in the acceptance and use of eHealth. 

Every block presented the newly added explanatory variable. It was found that the statistical significance in explained variance greatly increased by adding the performance expectancy to the model and then slightly by adding effort expectancy and social influence, while marginally after the facilitating conditions were added. This research presented that the acceptance and use of eHealth applications were mainly explained by performance expectancy, effort expectancy, and social influence, while no effect for facilitating conditions was found.

## 4. Discussion 

This study has several major findings and merits. This study’s overall finding is that participants aged 60–69 are quite open-minded toward eHealth, as three-fourths of the participants declared the acceptance and use of eHealth. Moreover, this study confirms the applicability of the UTAUT model in the context of eHealth among older adults in Poland, and thus, it establishes a framework that identifies the factors affecting the acceptance and use of eHealth. It was found that the acceptance and use of eHealth applications by respondents were strongly explained first by performance expectancy, then by effort expectancy and social influence, while no effect for facilitating conditions was found.

Consequently, this study validates the Unified Theory of Acceptance and Use of Technology (UTAUT) model in the context of healthcare and older adults in Poland, where similar research has not been undertaken. Thus, this study believes that the application of the UTAUT model makes a contribution to the literature and healthcare policy in that it is the first one to investigate the factors influencing eHealth adoption by older adults in Poland. By doing so, the theoretical gap in the acceptance of healthcare applications by older adults has been filled. Prior studies on eHealth focussed mainly on the level of using eHealth by older adults in Poland or by the population as a whole [68,69,71,72,73]. Moreover, this study would help government carries out feasible plans to facilitate the adoption of such technologies.

Such a result is consistent with most studies employing the UTAUT model but is contrary to a few of them. A similar survey conducted [76] in the Netherlands in May 2013 also showed that expected performance and effort were highly related to the acceptance and use of e-Health. However, they found that social influence was not an important factor, and facilitating conditions appeared to be important, which is completely opposite to the results of this study. It could be due to different cultures, as the Dutch culture could be more independent and not influenced by others. Moreover, this research included a different range of older adults aged between 57 and 77. In addition, the results of this study are similar to the results of [57], who found a positive influence of performance expectancy, effort expectancy, and social influence and no effect of facilitating conditions on the adoption of mHealth by older adults (sample of people aged 60 and above) in Bangladesh.

This study reported performance expectancy to be of significant importance, which indicates that the older adults’ acceptance and use of eHealth are influenced by their expectation of its usefulness. Thus, this finding is consistent with the previous studies employing the UTAUT model, highlighting that users were highly affected by the positive influence of the perceived benefits of the respective technologies/forms of eHealth investigated. However, these studies differed in terms of the type of analyzed technology (telemonitoring, telehealth, mHealth, etc.), the country where the research was conducted, and the scope of the older adults covered by the research [58,60,98,99,100,101,102].

The results of this study also showed that effort expectancy and social influence are important but minor factors influencing users’ acceptance of eHealth. With respect to effort expectancy [102] reported that the results of many empirical studies are inconclusive and differ according to the type of analyzed health technologies. In [60], it was found that effort expectancy in most of the analyzed studies had a negative impact on the adoption of health technologies. However, the research of [58,99,100,103] presented the importance of effort expectancy, but [98] showed the unimportance of it for older Chinese adults. Both [102] and [60] presented a quantity of research reporting the positive impacts of social influence on users’ acceptance of technology. Social influence also appeared to be quite an important factor in influencing users’ adoption of particular health technologies in the research of [58,98,99,101], but it had no importance in the research of [104]. These studies differ in the type of technology analyzed, and the country studied, and the obtained results are diverse. 

In contrast, facilitating conditions do not have a significant influence on the user’s acceptance of eHealth. This might be due to the fact that participants of the studies were in middle age when the Internet came to maturity in Poland. Thus, they are familiar with the use of the Internet. This result is opposite to the research of [58,103,105], while this same relationship was found by [99,100]. Moreover, [102] found inconclusive results regarding facilitating conditions that vary according to the type of health technology. In [60], it was found that the overall facilitating conditions were reported to positively impact seniors’ willingness to use health information technologies in most of the analyzed studies. However, the range of facilitating conditions differs among studies, and such differences might be due to the way facilitating conditions were operationalized.

This study has both theoretical and practical implications in the area of eHealth acceptance and uses in Poland. It contributes to the broad adoption literature by examining the applicability of the UTAUT model in the context of older adults in Poland. Overall, the results suggest that the UTAUT model provides a reasonable explanation for older adults’ acceptance and use of eHealth. To date, knowledge about the major acceptance factors for eHealth by older adults is limited in Poland. To the best knowledge of the author, this study is the first attempt to apply the constructs of the original UTAUT model and thus extensively explores factors that influence older adults’ acceptance and use of eHealth in Poland. With the growing demand for eHealth, evaluating the roles of the factors influencing adoption is a critical step toward defining success or failure with eHealth among older adults. In general, the current study not only points to the factors and possible barriers to eHealth acceptance and use by older adults but also emphasizes the positive aspects of and chances for eHealth implementation by older adults. 

The empirical findings could also have practical implications as they also show the ability of such research to provide practical guidance for the successful implementation of eHealth services among older adults. The results of this study disclose the significant role of the subsequent construct, such as performance expectancy, effort expectancy, and social influence to determine the acceptance and use of eHealth by older adults. With the proposed model, it would be possible to develop better eHealth services to meet the needs of older adults. These findings provide valuable information to eHealth technologies developers, service providers, and health policymakers to create better strategies and policies to endorse the acceptance and use of eHealth by older adults and to ensure the implementation of successful eHealth services.

The results show that performance expectancy is the most important factor, and therefore, manufacturers and product/service developers should emphasize the pragmatic functions and benefits of such systems that improve the usability of eHealth. The proper information/marketing campaign is also important as manufacturers should show that using health technology is an easy way to contact a doctor, that it works well, and that it is a convenient and pleasant way to interact, as well as to show the potential of these applications in fostering longevity. At this same time, the government should also popularize eHealth’s usefulness through social campaigns and let more people know about its convenience.

The importance of effort expectancy suggests that producers should concentrate more on the functionality of eHealth technologies/ applications for older adults in the age between 60 and 69 by ensuring that they have features that increase functionality, i.e., as shown by this research-making, a given technology easy to use and learn to use. For example, producers could include a broader and more accurate representation of users and thus take into account a wider range of their requirements regarding the ease of use of these technologies. The above stakeholders can even make these older adults into early users to reduce the unfriendly situations caused by the design of a particular technology. In this way, it is more likely to improve older adults’ acceptance and use of technology. Thus the advice of these older adults could be taken into consideration when designing any eHealth technology. Moreover, older adults could obtain some support in their use of eHealth technology. 

This study observes the relationship between social influence and technology acceptance and use. To achieve wider adoption of eHealth, manufacturers and application developers should consider approaches that exploit social influence among users. To promote social influence, the concerned policymakers may organize forums for sharing best-use practices, introduce champions (someone who could be the authority figure for older adults and would be able to influence others to accept the technology) who are motivated about diffusing awareness and the benefits of the system for generating positive word of mouth.

Consideration of the specific barriers and facilitators that influence the acceptance and use of eHealth by older adults is critical to improve the wider adoption and use of eHealth programs and to ensure the success of eHealth applications. The findings shed light on how valuable it would be to be able to carry out studies covering the entire population of specific groups of people, especially all old adults. Thus, service providers, health technology producers, planners, and policymakers could derive valuable information to develop strategies and policies for the successful implementation and acceleration of the adoption of this technology among older adults in Poland. The healthcare systems can be properly strengthened through the application of the UTAUT model—especially with respect to developing eHealth for older adults—and it would create an opportunity to protect people from unintended health inequity.

### Limitations and Future Study

The present study has several limitations that should be addressed in future studies. First, this study is conducted based on a representative sample (in the sense of gender and geographical locations) of older adults aged 60–69. Therefore, future research could cover larger populations of older adults and also in the age above 69. On the other hand, this research provides arguments for the importance of the creation of a wide database at the national level, which would make it possible to conduct detailed research on the usage of eHealth by older adults and others people in Poland. 

Second, this study employed the UTAUT model; therefore, future studies could employ more predictors, additional constructs, or an extended version of the UTAUT model [106] to further investigate the acceptance and use of eHealth by older adults. For example, the role of moods in the acceptance of eHealth as performance expectancy and effort expectancy could depend on the mood currently experienced by people.

Then, this study is limited with regard to the technologies which are under the term of eHealth. Thus findings are generalized to all technologies under the term eHealth. Therefore a similar study could be conducted for each separate technology to further verify the obtained results. In [107], it was proven that the type of technology/product can moderate the acceptance and use of particular devices or applications. This study could not include all of the eHealth technologies (e.g., robots) or other health information technologies; therefore, future research could be extended in such directions.

Further research would extend the current study to include additional demographic factors such as income and other variables to uncover a more generalized view of the proposed model in the context of Poland. Therefore, additional economic and cultural effects on the acceptance and use of eHealth should be explored to achieve possible higher predicting ability.

Such a set of studies would allow for the full generalization of the obtained results. All the limitations of this study may reveal opportunities for future academic research on the acceptance and use of eHealth among older adults.

## 5. Conclusions

This paper focused on the factors influencing the acceptance and use of eHealth by older adults. The results revealed that these three variables of the UTAUT model, such as performance expectancy, effort expectancy, and social influence, were important determinants explaining eHealth acceptance and use by Polish older adults. Performance expectancy was the strongest factor influencing the acceptance and use of eHealth; however, facilitating conditions appeared to not have a significant role in directly predicting the acceptance and use of older adults toward eHealth. Thus, the findings filled the research gap of older adults’ acceptance and use of eHealth in Poland and could help practitioners carry out feasible plans to facilitate the adoption of such technologies. 

## Figures and Tables

**Table 1:** Correlation matrix.

Elements of UTAUT Model	Performance Expectancy	Effort Expectancy	Social Influence	Facilitating Conditions
Performance expectancy				
Effort expectancy	0.60			
Social influence	0.65	0.44		
Facilitating conditions	0.23	0.41	0.14	
Acceptance and use of eHealth	0.58	0.54	0.44	0.25

Source: own calculation.

**Table 2:** The acceptance and use of eHealth.

Type of Respondents Answer:	% of All Respondents	% of All Women	% of All Men
Yes, definitely	27.50	14.00	13.50
Yes, probably	45.00	24.50	20.50
I do not know	16.25	10.50	5.75
Probably not	5.50	2.25	3.25
Definitely not	5.75	2.75	3.00

Source: own calculation.

**Table 3:** Perceptions of using eHealth application by respondents.

Four Elements of the UTAUT Model and Their Measures	Strongly Agree	Agree	I Do Not Know	Disagree	Strongly Disagree
***Performance expectancy: ***					
makes it easier to contact a doctor when it is needed,	15.25%	55.25%	18.25%	8.75%	2.50%
it makes it possible for me to live longer (to facilitate disease prevention and regular health monitoring),	9.75%	42.75%	28.75%	15.75%	3.00%
it works (functions) well,	4.25%	38.00%	40.25%	13.75%	3.75%
it is a pleasant way to interact.	8.00%	47.50%	23.75%	17.25%	3.50%
***Effort expectancy:***					
is easy to learn,	29.25%	54.25%	17.50%	2.25%	0.75%
fits easily into my daily routine,	15.25%	50.00%	21.25%	12.00%	1.50%
is easy to do.	24.50%	53.75%	15.50%	4.50%	1.75%
***Social influence: ***					
is something my family or friends do					
or would like to do.	7.75%	37.50%	30.00%	18.00%	6.75%
***Facilitating condition:***					
how easy or difficult do you find it to use the Internet	very difficult 0.25%	difficult 3.00%	neutral 13.50%	easy 40.75	very easy 42.00%*

* and 0.50% of respondents answered “I do not know”, and none answered, “I don’t use the Internet”. Source: own calculation.

**Table 4:** Nested regression analysis, presenting the acceptance and use of eHealth (n = 400).

	Block 1	Block 2	Block 3	Block 4	Final Model
R^2^	0.0191	0.3107	0.3387	0.3501	0.351
Change in R^2^		0.2855	0.0294	0.0115	0.0035
Sign of R^2^ change	<0.02163	<0.0000	<0.0000	<0.0000	<0.0000
Independent variables added	Beta	Beta	Beta	Beta	Beta
Gender					
-Female (ref)					
-Male					
Educational level					
-high (ref)					
-medium					
-vocational					
-low	0.15 *	0.13 *	0.10 *	0.10 *	0.09 *
Performance expectancy		0.54 **	0.41 **	0.33 **	0.33 **
Effort expectancy			0.21 **	0.20 **	0.18 *
Social influence				0.14 *	0.15 *
Facilitating conditions					

* *p* value < 0.05. ** *p* value < 0.001. Source: own calculation.

## Data Availability

The data presented in this study are available on request from the author.
